# Imaging and physician visits at cancer diagnosis: COVID ‐19 pandemic impact on cancer care

**DOI:** 10.1002/cam4.5321

**Published:** 2022-09-29

**Authors:** Rui Fu, Rinku Sutradhar, Qing Li, Timothy P. Hanna, Kelvin K. W. Chan, Natalie Coburn, Julie Hallet, Antoine Eskander, Pandemic–Ontario Collaborative in Cancer Research (POCCR)

**Affiliations:** ^1^ ICES Toronto Ontario Canada; ^2^ Institute of Health Policy, Management, and Evaluation University of Toronto Toronto Ontario Canada; ^3^ Department of Otolaryngology – Head and Neck Surgery Sunnybrook Research Institute, University of Toronto Toronto Ontario Canada; ^4^ Division of Cancer Care and Epidemiology, Cancer Research Institute, Queen's University Kingston Ontario Canada; ^5^ Ontario Institute for Cancer Research (OICR) Toronto Ontario Canada; ^6^ Odette Cancer Centre – Sunnybrook Health Sciences Centre Toronto Ontario Canada; ^7^ Ontario Health – Cancer Care Ontario Toronto Ontario Canada; ^8^ Department of Surgery University of Toronto Toronto Ontario Canada

**Keywords:** cancer diagnosis, COVID‐19, diagnostic imaging, telemedicine, ultrasound

## Abstract

**Background:**

Little is known about the COVID‐19 pandemic impact on the provision of diagnostic imaging and physician visits at cancer diagnosis.

**Methods:**

We used administrative databases from Ontario, Canada, to identify MRI/CT/ultrasound scans and in‐person/virtual physician visits conducted with cancer patients within 91 days around the date of diagnosis in 2016–2020. In separate segmented regression procedures, we assessed the trends in weekly volume of these services per thousand cancer patients in prepandemic (June 26, 2016 to March 14, 2020), the change in mean volume at the start of the pandemic, and the additional change in weekly volume during the pandemic (March 15, 2020, to September 26, 2020).

**Results:**

Totally, 403,561 cancer patients were included. On March 15, 2020 (COVID‐19 arrived), mean scan volume decreased by 12.3% (95% CI: 6.4%–17.9%) where ultrasound decreased the most by 31.8% (95% CI: 23.9%–37.0%). Afterward, the volume of all scans increased further by 1.6% per week (95% CI: 1.3%–2.0%), where ultrasound increased the fastest by 2.4% (95% CI: 1.8%–2.9%). Mean in‐person visits dropped by 47.4% when COVID‐19 started (95% CI: 41.6%–52.6%) while virtual visits rose by 55.15‐fold (95% CI: 4927%‐6173%). In the pandemic (until September 26, 2020), in‐person visits increased each week by 2.6% (95% CI: 2.0%–3.2%), but no change was observed for virtual visits (*p* ‐value = 0.10).

**Conclusions:**

Provision of diagnostic imaging and virtual visits at cancer diagnosis has been increasing since the start of COVID‐19 and has exceeded prepandemic utilization levels. Future work should monitor the impact of these shifts on quality of delivered care.

## Graphical abstract

In Ontario, Canada, the provision of diagnostic imaging and virtual physician visits at cancer diagnosis has been increasing since the start of COVID‐19 and has already exceeded prepandemic weekly utilization levels, as of September 26, 2020. Future work should monitor the impact of these shifts on quality of delivered care.

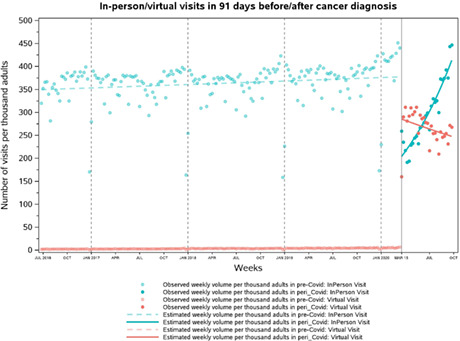

## 1 INTRODUCTION

The COVID‐19 pandemic has adversely impacted many aspects of cancer care.^1^ Pandemic‐related delays in cancer diagnosis and treatment have been reported.^2^, ^3^, ^4^, ^5^, ^6^ However, little work has been done to assess the impact of COVID‐19 on essential care services, such as imaging and physician visits, that are used for diagnosing, staging, and care planning.^7^ Changes in their use may impact treatment decision making, quality of care, long‐term patient outcomes, and costs of care. To minimize repercussions for patients and to prepare the system to mitigate future waves of the pandemic, we need to understand how these services responded to the pandemic.

The shift toward diagnostic imaging may depend on modality, as computed tomography (CT), magnetic resonance imaging (MRI), and ultrasound scans require different levels of physical contact between patients and health professionals and thus might show varied changes in utilization during the pandemic.^8^, ^9^ The desire to reduce physical contact while achieving care goals also gives rise to a surge in virtual visits in the general patient population.^10^, ^11^, ^12^, ^13^, ^14^, ^15^ However, no study has quantified this phenomenon in the initial management of cancer patients. As COVID‐19 continues to evolve and negatively affect the cancer system, combining virtual consultations with imaging for diagnosis and staging purposes might represent a feasible solution to combat the current staff shortage and adhere to physical distancing policy. Hence, in this study, we aimed to retrospectively examine how a large provincial cancer system responded to COVID‐19 by adjusting the use of diagnostic imaging and virtual visits in managing new cancer patients. These results will illustrate how combining these care methods could maintain appropriate cancer diagnosis and management in further waves of COVID‐19 and other future health crises.

## 2 METHODS

### 2.1 Data sources

This retrospective longitudinal time series study used linked, population‐based, administrative data from Ontario, Canada (population ~14.8 million). A cohort of cancer patients covered by the provincial health care program (OHIP) is available through Ontario Cancer Registry (OCR), which has up to 98% capture rate of cancer incidences across the province.^16^, ^17^ At the time of this analysis, the OCR had reliable data until September 26, 2020. Diagnostic imaging and visits were determined using physician billing records from OHIP.^18^ Patient sex and age at cancer diagnosis were obtained from Ontario's Registered Persons Database. The Immigration, Refugees and Citizenship Canada (IRCC) Permanent Resident Database (with data from January 1985 to May 2017) was used to identify individuals who immigrated to Ontario during that period. Urban living was determined from Statistics Canada's Postal Code Conversion File.^19^ Comorbidity was represented by the Elixhauser Comorbidity Index using hospitalization records over 5 years before the date of cancer diagnosis.^20^ Material deprivation and ethnic concentration were both derived from the Ontario Marginalization Index database. Material deprivation reflects the proportion of a population that is without a high school degree, families that are lone‐parent, receiving government transfer payments, unemployed, low‐income, and living in dwellings that are in need of major repair.^21^ Ethnic concentration measures the proportion of a population that is a recent immigrant and self‐identified as visible minority.^21^ These data sets were linked using unique encoded identifiers and analyzed at ICES. The use of the data in this study is authorized under section 45 of Ontario's Personal Health Information Protection Act (PHIPA) and does not require review by a Research Ethics Board.

### 2.2 Study cohort

Following our previous works that examined the impact of COVID‐19 on cancer incidence, surgery, and emergency department visits,^3^, ^22^, ^23^ we used March 15, 2020, to mark the start of pandemic control measures as hospitals across the province were advised to halt nonemergent or elective procedures.^24^ As such, we defined a 222‐week accrual window for diagnostic imaging and physician visits that comprised a pre‐pandemic period (194 weeks from June 26, 2016, to March 14, 2020) and a pandemic period (28 weeks from March 15, 2020, to September 26, 2020). We identified cancer diagnoses from the OCR using the International Statistical Classification of Diseases and Related Health Problems, Tenth Revision (ICD‐10), O.3 codes (see Table S1). For each week of the accrual window, we counted a diagnostic imaging or visit to the numerator if (i) the patient was 18 years of age at the start of the week and (ii) this procedure occurred around the date of a cancer diagnosis, i.e., it happened within a 183‐day window that included the date of diagnosis and 91 days before and after this date. If a procedure can be attributed to multiple cancer diagnoses, we only counted this procedure once in the numerator.

### 2.3 Outcome

We focused on diagnostic imaging (including CT, MRI, and ultrasound scans) and physician visits (including in‐person visits and virtual visits) performed around the date of cancer diagnosis. We reported the weekly volume of these services per 1000 cancer patients. We did not include positron emission tomography (PET) due to its narrow indication for cancer diagnosis and staging in Ontario.^25^ Furthermore, cancer patients who are eligible for publicly funded PET scans are required to receive CTs or MRIs prior to approval.^25^ Virtual visits initially comprised videoconferencing visits delivered through Ontario Telemedicine Network (OTN)'s platform and e‐assessments which are electronic communications between physicians with certain specialty to discuss patient treatment; since March 14, 2020, virtual visits also included phone or video visits using non‐OTN platform as temporary codes became available for physicians to claim under an emergency order.^26^ Back billing was not allowed and these codes are set to expire on September 30, 2022.^27^ On April 1, 2020, the B‐codes that physicians can previously bill to receive a $35 premium for establishing a new patient encounter on OTN and a $15 premium for each subsequent OTN visit were discontinued.^28^

### 2.4 Statistical analysis

We compared the distributions of characteristics among patients in the cohort whose cancer diagnosis occurred during pre‐pandemic vs. the pandemic era. We used standardized differences greater than 0.1 to indicate a statistically significant imbalance.^29^ Segmented negative binomial regression analyses were carried out independently for diagnostic imaging and physician visits to estimate trends in weekly volume per 1000 cancer patients. This analytical method has been previously employed by our group to study the temporal trends in the provision of other healthcare activities during the pandemic.^3^, ^10^, ^22^ We were specifically interested in obtaining estimates for the following: the weekly pre‐pandemic volume trend (slope); the immediate change in mean volume at the start of the pandemic (relative change in intercept), and the subsequent change in weekly volume during the pandemic (change in slope). A detailed description of this regression model is presented in Supplemental Methods. Analyses were 2‐sided where *p*‐value<0.05 signaled statistical significance. We performed all analyses using SAS Enterprise Guide version 7.15 (SAS Institute).

## 3 RESULTS

### 3.1 Descriptive analysis

Table 1 presents the 403,561 cancer patients in the study cohort, stratified by whether the diagnosis occurred pre‐pandemic (*n* = 362,085, 89.7%) or in the pandemic (*n* = 41,476, 10.3%). Overall, we did not identify any statistically significant imbalance of characteristics between the two groups of patients.

**TABLE 1:** Characteristics of patients diagnosed with cancer before and after the start of COVID‐19

Characteristics	Cancer diagnosis in pre‐pandemic (*n* = 362,085)	Cancer diagnosis in the pandemic (*n* = 41,476)	Standardized difference
Age at diagnosis, mean ± SD, y	64.41 ± 16.00	64.90 ± 15.47	0.03
Sex, female	194,678 (53.8%)	21,944 (52.9%)	0.02
Immigrant	42,824 (11.8%)	4979 (12.0%)	0.01
Urban residence^a^	315,382 (87.1%)	35,919 (86.6%)	0.01
Regions of residence^a^			
Central	103,572 (28.6%)	11,751 (28.3%)	0.01
East	93,507 (25.8%)	10,821 (26.1%)	0.01
North	25,545 (7.1%)	3013 (7.3%)	0.01
Toronto	30,348 (8.4%)	3212 (7.7%)	0.02
West	108,984 (30.1%)	12,661 (30.5%)	0.01
Material deprivation quintile^a^ ^,^ ^b^			
1 (most privileged)	77,940 (21.5%)	9188 (22.2%)	0.02
2	74,000 (20.4%)	8484 (20.5%)	0
3	69,797 (19.3%)	8036 (19.4%)	0
4	68,830 (19.0%)	7795 (18.8%)	0.01
5 (most deprived)	68,306 (18.9%)	7588 (18.3%)	0.01
Ethnic concentration quintile^a^ ^,^ ^c^			
1 (lowest concentration)	75,850 (20.9%)	8996 (21.7%)	0.02
2	71,522 (19.8%)	8158 (19.7%)	0
3	68,006 (18.8%)	7845 (18.9%)	0
4	69,097 (19.1%)	7829 (18.9%)	0.01
5 (highest concentration)	74,398 (20.5%)	8263 (19.9%)	0.02
Elixhauser comorbidity grouping^d^			
0	37,121 (10.3%)	4428 (10.7%)	0.01
1	29,936 (8.3%)	3749 (9.0%)	0.03
2	22,071 (6.1%)	2586 (6.2%)	0.01
3 or above	31,522 (8.7%)	3533 (8.5%)	0.01
No Hospitalization	241,435 (66.7%)	27,180 (65.5%)	0.02
Cancer site			
Breast	45,629 (12.6%)	5153 (12.4%)	0.01
Central nervous system	3987 (1.1%)	542 (1.3%)	0.02
Cervical	29,993 (8.3%)	2763 (6.7%)	0.06
Colorectal	36,932 (10.2%)	4702 (11.3%)	0.04
Endocrine	11,958 (3.3%)	1266 (3.1%)	0.01
Esophagus	3401 (0.9%)	491 (1.2%)	0.02
Genitourinary	31,449 (8.7%)	3595 (8.7%)	0
Gynecological (excluding cervix)	18,247 (5.0%)	2310 (5.6%)	0.02
Head and neck	10,293 (2.8%)	1228 (3.0%)	0.01
Hepatic, pancreatic or biliary	15,829 (4.4%)	2068 (5.0%)	0.03
Lung	39,736 (11.0%)	4701 (11.3%)	0.01
Lymphoma	16,983 (4.7%)	1757 (4.2%)	0.02
Melanoma	16,873 (4.7%)	1828 (4.4%)	0.01
Ophthalmic	151 (0.0%)	15 (0.0%)	0
Paraneoplastic neurologic syndromes	43 (0.0%)	8 (0.0%)	0.01
Prostate	35,392 (9.8%)	3713 (9.0%)	0.03
Sarcoma	5536 (1.5%)	732 (1.8%)	0.02
Skin	1195 (0.3%)	97 (0.2%)	0.02
Stomach	5733 (1.6%)	719 (1.7%)	0.01
Other	32,725 (9.0%)	3788 (9.1%)	0

*Note*: We captured a cancer diagnosis if any part of its 183‐day window (i.e., 91 days around the date of diagnosis) fell into the accrual window (June 26, 2016 to September 26, 2020). A diagnosis was considered to occur in pre‐pandemic if the date of diagnosis was before March 15, 2020, otherwise it happened in the pandemic period. Cancer diagnoses were restricted to one per person per day. If a diagnostic imaging procedure fell into the 183‐day window of multiple cancer diagnoses, we attributed it to the first diagnosis in order to generate descriptive data for the distribution of cancer site.

Abbreviations: SD, standard deviation; yr, year;

^a^ Missing data were omitted from the descriptive analysis. In total, less than 1.0% of data were absent. Missing data did not differ between the two groups of patients since all standardized differences were smaller than 0.01.

^b^ Material deprivation was derived from the Ontario Marginalization Index. It captures neighborhood characteristics, including educational attainment, unemployment rates, single‐parent families, low‐income households, conditions of dwellings and families receiving government transfer payments.

^c^ Ethnic concentration was obtained from the Ontario Marginalization Index. It measures the proportion of a population that is a recent Canadian immigrant (i.e., having obtained a landed immigrant or permanent resident status up to 5 years) and self‐identified as visible minority.

^d^ The Elixhauser comorbidity grouping was calculated using a 5‐year look‐back window in administrative data for any hospitalization.

### 3.2 Impact of COVID‐19 on weekly volume of diagnostic imaging around cancer diagnosis

There was a 18.8% relative increase in volume of any scans after the start of COVID‐19, from 133.2 to 158.2 per 1000 patient‐weeks. MRI scans showed the greatest increase (26.7%, from 13.1 to 16.6 per 1000 patient‐weeks), followed by CT scans (22.3%, from 99.1 to 121.2 per 1000 patient‐weeks). Volume of ultrasound declined by 2.4%, from 21.0 to 20.5 per 1000 patient‐weeks.

**FIGURE 1:**
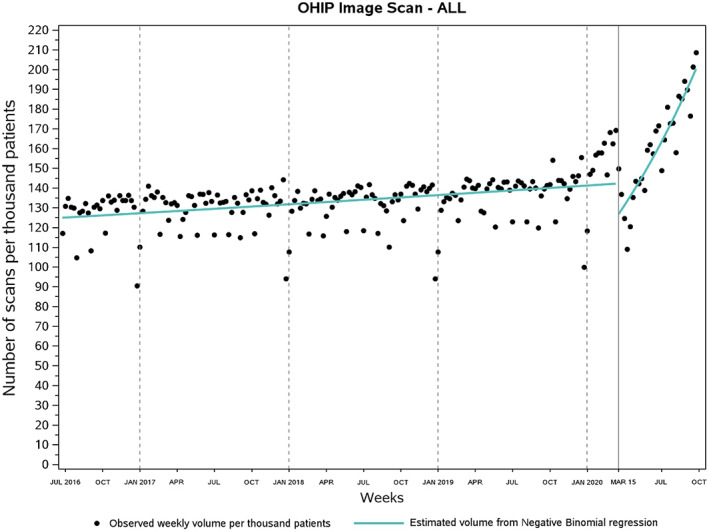
Weekly use of diagnostic imaging around the date of diagnosis per thousand cancer patients in Ontario, Canada. On average, weekly volume of scans per 1000 cancer patients rose by 18.8% from pre‐pandemic (133.2; June 26, 2016 to March 14, 2020) to the pandemic era (158.2; March 15, 2020 to September 26, 2020). Volume of scans returned to prepandemic weekly utilization levels during the week of May 10–16, 2020. OHIP, Ontario Health Insurance Plan.

**FIGURE 2:**
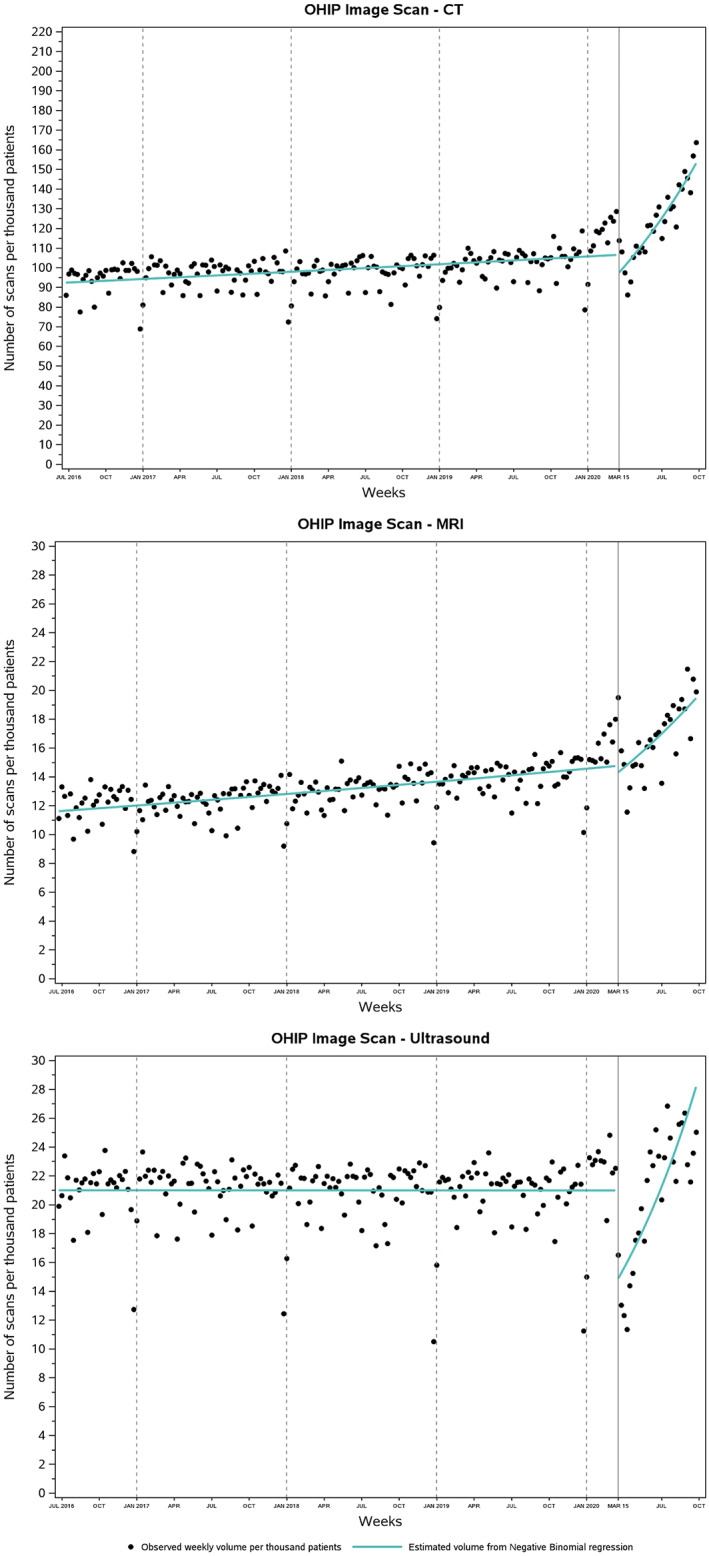
Weekly use of diagnostic imaging stratified by type. Weekly volume of CT, MRI, and ultrasound per 1000 cancer patients recovered to pre‐pandemic levels in April 26–May 2, April 12–18, and June 28–July 4, 2020, respectively. OHIP, Ontario Health Insurance Plan.

According to the regression analysis (Table 2), during the pre‐pandemic period, total volume of scans per 1000 cancer patients was marginally increasing by 0.07% for each subsequent week (rate ratio [RR], 1.0007; 95% confidence interval [CI], 1.0005–1.0009). Specifically, the volume of CT scans (RR: 1.0007; 95% CI, 1.0005–1.0009) and MRI scans (RR, 1.0012; 95% CI: 1.0010–1.0015) rose by 0.07% and 0.12% per week, respectively, but the volume of ultrasound remained stable over weeks (RR, 1.0000; 95% CI, 0.9997–1.0003). On March 15, 2020, there was a 12.3% reduction in scan mean volume (RR, 0.877; 95% CI, 0.821–0.936). The largest mean volume reduction was observed for ultrasound (RR, 0.692; 95% CI, 0.630–0.761), followed by CT scans (RR, 0.901; 95% CI, 0.844–0.960). No difference was observed for MRI scans (RR, 0.960; 95% CI, 0.892–1.033). Afterward, during the pandemic period, the volume of scans increased by 1.6% per week (RR, 1.016; 95% CI, 1.013–1.020) and ultimately exceeded pre‐pandemic levels after the week of May 10–16, 2020 (Figure 1). The fastest weekly volume increase during the pandemic was for ultrasound (RR, 1.024; 95% CI, 1.018–1.029), followed by CT scans (RR, 1.016; 95% CI, 1.012–1.020), and finally, MRI scans (RR, 1.010; 95% CI, 1.006–1.014; Figure 2). Ultrasound, CT, and MRI utilization reached pre‐pandemic levels during the week of June 28–July 4, April 26–May 2, and April 12–18, 2020, respectively.

**TABLE 2:** Segmented negative binomial regression analysis on weekly volume of diagnostic imaging per thousand cancer patients around date of cancer diagnosis in Ontario, Canada

Diagnostic imaging type	Relative change in weekly volume (slope before the start of COVID‐19)	Relative change in mean volume at the start of COVID‐19 (relative change in intercept)	Relative change in weekly volume (further change in slope after the start of COVID‐19)
All scans	1.0007	0.877	1.016
	(1.0005–1.0009)	(0.821–0.936)	(1.013–1.020)
	*p*‐value <0.01	*p*‐value <0.01	*p*‐value <0.01
CT scans	1.0007	0.901	1.016
	(1.0005–1.0009)	(0.844–0.960)	(1.012–1.020)
	*p*‐value <0.01	*p*‐value <0.01	*p*‐value <0.01
MRI scans	1.0012	0.960	1.010
	(1.0010–1.0015)	(0.892–1.033)	(1.006–1.014)
	*p*‐value <0.01	*p*‐value = 0.27	*p*‐value <0.01
Ultrasound scans	1.0000	0.692	1.024
	(0.9997–1.0003)	(0.630–0.761)	(1.018–1.029)
	*p*‐value = 0.98	*p*‐value <0.01	*p*‐value <0.01

*Note*: We use March 15, 2020, to represent the start of the COVID‐19 pandemic in Ontario, Canada, as hospitals across the province were advised to halt non‐emergent/elective procedures. Pre‐pandemic period includes 194 weeks from June 26, 2016, to March 14, 2020, while the pandemic period includes 28 weeks from March 15, 2020, to September 26, 2020. For each parameter, we report the ratio, the associated 95% confidence interval (in parenthesis) and the *p*‐value testing whether the ratio equals to 1.The regression coefficients for ‘all scans’ can be interpreted as followed: the total volume of scans per thousand patients was increasing at 0.07% for each week during pre‐pandemic, followed by a drop in the mean volume of 12.3% during the start of the pandemic, then a weekly rise of 1.7% (i.e., the product of pre‐pandemic slope, 1.0007, and the relative change in slope in pandemic vs. pre‐pandemic era, 1.016) until September 26, 2020. A detailed description of the segmented regression model is presented in Supplemental Methods.

Abbreviations: CT, computerized tomography; MRI, magnetic resonance imaging.

### 3.3 Impact of COVID‐19 on weekly volume of physician visits around cancer diagnosis

Total volume of visits rose by 51.9% in the pandemic relative to pre‐pandemic levels, from 366.2 to 556.1 per 1000 patient‐weeks. The volume of in‐person visits dropped by 20.3% (from 362.7 to 288.9 per 1000 patient‐weeks). Meanwhile, the volume of virtual visits surged from 3.5 to 267.2 per 1000 patient‐weeks, resulting in a 7534.3% relative increase. OTN visits declined by over 70% (from 3.4 to 1.0 per 1000 patient‐weeks) while e‐assessments remained rare throughout the study period (from 0.04 to 0.05 per 1000 patient‐weeks).

**FIGURE 3:**
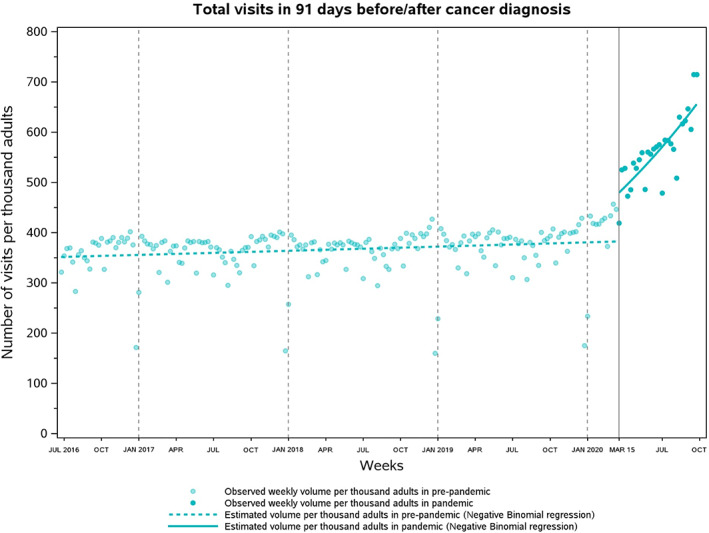
Weekly volume of physician visits conducted around date of cancer diagnosis per thousand cancer patients in Ontario, Canada. On average, weekly volume of physician visits per 1000 cancer patients rose by 51.9% from pre‐pandemic (366.2; June 26, 2016 to March 14, 2020) to the pandemic era (556.1; March 15, 2020 to September 26, 2020).

**FIGURE 4:**
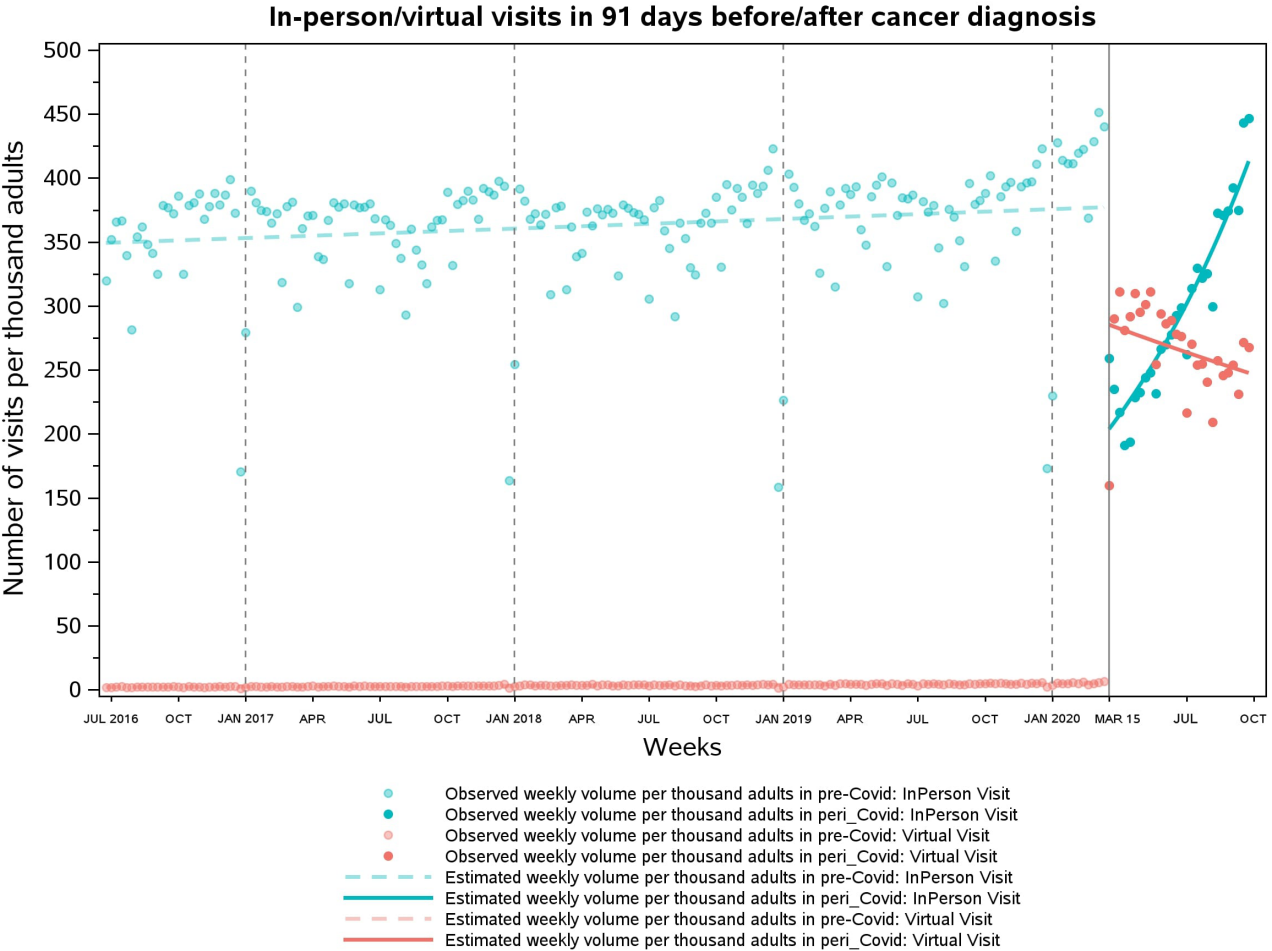
Weekly volume of in‐person and virtual visits conducted with adult cancer patients around date of diagnosis per 1000 cancer patients in Ontario, Canada. Virtual visits comprised both OTN visits and e‐assessments during prepandemic and later also included phone visits when phone codes were launched under an emergency order. On average, there was a 20.3% decline in weekly volume per 1000 patients for in‐person visits (from 362.7 to 288.9) and a 7534.3% relative increase in weekly volume of virtual visits (from 3.5 to 267.2). Weekly utilization of in‐person visits returned to prepandemic levels during the week of August 30–September 5, 2020.

During pre‐pandemic era, the weekly volume of any visit per 1000 cancer patients followed a small but significant increasing trend (RR, 1.0004; 95% CI, 1.0001–1.0008; Table 3; Figure 3), since both in‐person and virtual visits (including OTN visits and e‐assessments) were increasing at 0.04% (RR, 1.0004; 95% CI, 1.00007–1.00072) and 0.46% (RR, 1.0046; 95% CI, 1.0042–1.0050) for each week (Figure 4). As COVID‐19 arrived on March 15, 2020, the mean volume of in‐person visits declined by 47.4% (RR, 0.526; 95% CI, 0.474–0.584) while that of virtual visits—now including non‐OTN phone/video visits—rose dramatically by over 55‐fold (RR, 55.15; 95% CI, 49.27–61.73). Overall, the mean volume of all visits increased by 24% (RR, 1.24; 95% CI, 1.12–1.38) at the start of the pandemic. During the pandemic, in‐person visits started to gradually increase by an additional 2.6% each week relative to pre‐pandemic levels (RR, 1.026; 95% CI, 1.020–1.032) and returned to pre‐pandemic weekly utilization during the last week of August 2020 (August 30–September 5, 2020). Meanwhile, virtual visits were not correspondingly decreasing (RR, 0.995; 95% CI, 0.989–1.001) due to the persistent high‐volume of phone/video visits (Figure S1). OTN visits declined by 74% per week in the pandemic (RR, 0.260; 95% CI: 0.214–0.317; Figure S2), while e‐assessments were stable over weeks (RR, 1.013; 95% CI: 0.976–1.052; Figure S3). The volume of all visits increased by 1.1% (RR, 1.011; 95% CI, 1.005–1.017) for each week in the pandemic.

**TABLE 3:** Segmented negative binomial analysis on weekly volume of physician visits per thousand cancer patients around date of cancer diagnosis in Ontario, Canada

Physician visits type	Relative change in weekly volume (slope before the start of COVID‐19)	Relative change in mean volume at the start of COVID‐19 (relative change in intercept)	Relative change in weekly volume (further change in slope after the start of COVID‐19)
All visits	1.0004	1.24	1.011
	(1.0001–1.0008)	(1.12–1.38)	(1.005–1.017)
	*p*‐value <0.01	*p*‐value <0.01	*p*‐value <0.01
In‐person visits	1.0004	0.526	1.026
	(1.00007–1.00072)	(0.474–0.584)	(1.020–1.032)
	*p*‐value = 0.02	*p*‐value <0.01	*p*‐value <0.01
Virtual visits	1.0046	55.15	0.995
	(1.0042–1.0050)	(49.27–61.73)	(0.989–1.001)
	*p*‐value <0.01	*p*‐value <0.01	*p*‐value = 0.10
OTN visits	1.0046	22.75	0.260
	(1.0041–1.0051)	(13.12–39.43)	(0.214–0.317)
	*p*‐value <0.01	*p*‐value <0.01	*p*‐value <0.01
E‐assessments	1.000	1.078	1.013
	(0.998–1.002)	(0.558–2.080)	(0.976–1.052)
	*p*‐value = 0.78	*p*‐value = 0.82	*p*‐value = 0.49

*Note*: Virtual visits comprised OTN visits and e‐assessments during the pre‐pandemic era and later also included phone/video visits once phone codes were launched under a temporary emergency order. The regression coefficients of ‘in‐person visits’ can be interpreted as followed: the volume of in‐person visits per thousand patients was increasing at 0.04% for each week during pre‐pandemic, followed by a drop in the mean volume of 47.4% at the start of the pandemic, then a weekly rise of 2.7% (i.e., the product of pre‐pandemic slope, 1.0004, and the relative change in slope in pandemic vs. pre‐pandemic era, 1.026) until September 26, 2020 (see Supplemental Methods for a detailed description).

Abbreviations: OTN, Ontario Telemedicine Network.

## 4 DISCUSSION

After an initial drop in volume, diagnostic imaging has recovered in the pandemic era resulting in an 18.8% relative increase of use among new cancer patients. The growth in virtual visits can be attributed to the introduction of new physicians claims for phone/video consultations and assessments which were required when restrictions were placed on in‐person visits. The high volume of peri‐diagnosis virtual visits persisted even when in‐person visit volumes recovered to pre‐pandemic levels. These findings shed light on a potential practice shift in how physicians manage new cancer patients and different patient needs in the pandemic era.

Our findings are consistent with prior analyses that revealed the pandemic to have an immediate negative effect on the provision of cancer imaging.^7^, ^8^, ^9^, ^30^, ^31^, ^32^, ^33^ Furthermore, we found evidence on a potentially unique impact of the pandemic on ultrasound that require a close and sustained physical interaction between the radiology technician and the patient. As precautions were heightened, ultrasound would have become more costly in terms of time and resource allocated (due to the personal protective equipment), which might explain the greater drop in ultrasound than in CT or MRI.^9^, ^31^ These observations are concerning as ultrasound‐guided biopsy is more commonly used in cancer diagnosis (such as for liver and thyroid cancers) than CT or MRI‐guided biopsies; hence, if the pandemic has temporarily but significantly limited the use of ultrasound, a group of cancer patients may have missed the opportunity to receive timely treatment. This will be the focus of future work and was beyond the scope of this study.

Prior evidence has suggested CT and MRI volume to recover quickly to pre‐pandemic levels after the initial drop.^7^, ^8^, ^32^ In our study, CT and MRI use surpassed pre‐pandemic levels by 22.3% and 26.7%, as of September 2020. These results may indicate the success of provincial strategies to sustain CT/MRI operation amid the first wave of COVID‐19. Specifically, the 85 diagnostic CT/MRI facilities in Ontario were advised to expand operating hours and consider utilizing mobile or research scanners for additional capacity. To help patients in low‐capacity regions to access imaging services quickly, a provincially coordinated communication strategy was implemented to inform physicians and patients of alternative CT/MRI locations in their neighboring region.^34^ The rapid rise in CT/MRI use during the pandemic might represent attempts of radiologists to prioritize the care of cancer patients over noncancer patients and to catch up on missed workups. It may also mean that due to surgical delay, physicians requested another CT/MRI each time a surgery was being rescheduled; physicians may have relied more on imaging for staging and symptom assessment as they saw a decrease in patients attending physical examinations.^35^ We do not fully understand the extent to which imaging can adequately and safely replace the need for physical examination. This may also differ by cancer type and location. A deeper understanding of the decision‐making process and access to resource is required to elucidate the rationale for these shifts.

Single‐center studies have demonstrated the merit of virtual oncology care during the pandemic,^36^, ^37^ but no study to date has quantified the impact of the pandemic on visit volume. Our analysis confirms the parallel increase of similar magnitude in virtual care observed in new cancer patients as has been observed in the general patient population.^10^, ^11^, ^12^, ^13^, ^14^, ^15^ We also conclude the large increase in virtual visits was almost entirely attributed to phone/video visits reimbursed through the new codes,^10^, ^14^, ^15^ as exclusive use of OTN platform to conduct visits dropped dramatically when the premium codes expired. Taking into account our findings on an increased use of imaging in the pandemic era, this rise in virtual care may reflect a distinct shift in cancer care practice where physicians are more likely to request imaging rather than (or on top of) physical examination in conjunction with a virtual visit for cancer staging and symptom assessment. Since October 1, 2021, a tracking code is required to be billed with these codes to indicate the modality of the visit (phone or video).^27^ This new measure will generate data to examine the efficacy of using audio‐only phone visits with imaging to assess new cancer patients.

We found an increase in total physician visits provided to new cancer patients in the pandemic era, without evidence of population profile differences between patients diagnosed in the pandemic and pre‐pandemic era. An increase in mental health disorders experienced by cancer patients amid the pandemic may contribute to this rise in visits,^7^ and with the roll out of vaccines, patients may have reached out to physicians more frequently to seek their advice. Physicians might request more checkups with patients receiving unconventional non‐surgical first‐line cancer therapy to provide more oversight.^38^ This rise in virtual visits may also be related to the fact that cancer physicians have always been calling their patients in addition to their in‐person visits, but previously this was not billable through OHIP and simply may not have been captured. Since virtual care means more physicians can operate out of the same clinical space it is unlikely to raise infrastructure expenses^39^; however, the surge in virtual visits found in this study certainly leads to increased expenditures on physician billings.^40^ As such, our findings call for analyses to evaluate the incremental benefit of supplementing in‐person visits with virtual visits and whether this is cost effective to society.

These findings must be interpreted within the context of the study design. Unlike Zattra et al,^8^ we did not further consider a “post‐peak” period; instead, we followed a simplified practice common in the literature to define a pre‐pandemic era and a pandemic era using a single threshold.^3^, ^7^, ^10^, ^22^, ^23^, ^33^ Future study needs to include multiple thresholds that represent the onset of subsequent COVID‐19 waves, milestones in vaccination, and changes in social mitigating methods to provide a fulsome description on trends in cancer services utilization. Second, we did not explicitly consider the content of each procedure, although by restricting imaging and visits to be those delivered around the time of cancer diagnosis, we implicitly captured services that were very likely used for cancer diagnosis and staging purposes. A recently published report that identified CT/MRI for cancer diagnosis/staging and virtual cancer care for treatment consultation, follow‐up, and psychosocial support has provided similar findings with ours.^7^ Third, our findings may not be generalizable to jurisdictions without universal healthcare. Hence, researchers need to interpret our results with caution and potentially replicate this analysis using local data to guide policy decision making.

## 5 CONCLUSIONS

In this large retrospective analysis using administrative data in Ontario, Canada, we found diagnostic imaging and physician visits provided to patients at cancer diagnosis to be greatly impacted by the COVID‐19 pandemic. Despite an initial decrease in volume, provision of diagnostic imaging and virtual visits has been steadily increasing in the pandemic era, even exceeded pre‐pandemic utilization levels. These findings imply that in the era of physical distancing, combining virtual consultations with diagnostic imaging can be used to manage new cancer patients, although the cost‐effectiveness of this care combination to the healthcare system, its long‐term clinical implications to cancer care outcomes and patients' quality of life, and its sustainability to the society warrant follow‐up investigations. While virtual codes are here to stay, policymakers and clinicians need to ensure their appropriate use in managing cancer patients to prevent overuse while optimizing care quality.

## AUTHOR CONTRIBUTIONS

Eskander and Sutradhar had full access to the study data and take responsibility for the integrity of the data and the accuracy of the data analysis. Li was involved in the data analysis. Fu led the drafting of the manuscript. All authors were involved in study design, interpretation of the data, review and approval of the manuscript, and decision to submit the manuscript for publication.

## FUNDING INFORMATION

This work is supported by a Sunnybrook Research Institute and Sunnybrook Foundation COVID‐19 Response Grant and a Canadian Institutes of Health Research Operating Grant (#179892). The funders were not involved in any part of the study, including design and conduct of the study; collection, management, analysis, and interpretation of the data; preparation, review, or approval of the manuscript; and decision to submit the manuscript for publication.

## CONFLICT OF INTEREST

Dr. Antoine Eskander has the following disclosures: Research Funding: Merck (2019); Consultant: Bristol Myers (2019). All other listed authors have disclosed that they have not received any financial consideration from any person or organization to support the preparation, analysis, results, or discussion of this article.

## ETHICS APPROVAL/PATIENT CONSENT STATEMENT

The use of secondary deidentified administrative data provided by ICES in this project is authorized under section 45 of Ontario's Personal Health Information Protection Act (PHIPA) and thus does not require review by a Research Ethics Board even though individual written consent was not obtained.

## Supporting information


Appendix S1

## Data Availability

The dataset from this study is held securely in coded form at ICES. While legal data sharing agreements between ICES and data providers (e.g., healthcare organizations and government) prohibit ICES from making the dataset publicly available, access may be granted to those who meet pre‐specified criteria for confidential access, available at www.ices.on.ca/DAS (email: das@ices.on.ca). The full dataset creation plan and underlying analytic code are available from the authors upon request, understanding that the computer programs may rely upon coding templates or macros that are unique to ICES and are therefore either inaccessible or may require modification.
