# Supplementary material for: Imaging and physician visits at cancer diagnosis: COVID ‐19 pandemic impact on cancer care
Source: Cancer Med. 2022 Sep 29;12(5):6056–67. doi: 10.1002/cam4.5321 (PMC10028129; doi:10.1002/cam4.5321)
Supplement: Supplementary file 1 — Appendix S1 [file CAM4-12-6056-s001.docx]

**Imaging and physician visits at cancer diagnosis: COVID-19 pandemic impact on cancer care**

**Supplemental Table.** Identifying cancer types from the Ontario Cancer Registry (OCR)

| **Cancer site** | **ICD-O-3 code** |
| --- | --- |
| Breast | C50 |
| Central nervous system | C70.0, C70.1, C70.9, C71, C72 |
| Colorectal | C17, C18, C19.9, C20.9, C21.0, C21.1, C21.2, C21.8 |
| Cervical | C53.0, C53.1, C53.8, C53.9 |
| Endocrine | C73.9, C74.0, C74.1, C74.9, C75 |
| Esophagus | C15 |
| Genitourinary | C60, C62, C64, C65, C66, C67, C68 |
| Gynecological exclude cervical | C51, C52, C54, C55, C56, C57 |
| Head and neck | C10.0, C10.1, C10.2, C10.3, C10.4, C10.8, C10.9, C01.9, C02.0, C02.1, C02.2, C02.3, C02.4, C02.8, C02.9, C03.0, C03.1, C03.9, C04.0, C04.1, C04.8, C04.9, C05.0, C05.1, C05.2, C05.8, C05.9, C06.0, C06.1, C06.2, C06.8, C06.9, C07.9, C08.0, C08.1, C08.8, C08.9, C09.0, C09.1, C09.8, C09.9, C11.0, C11.1, C11.2, C11.3, C11.8, C11.9, C12.9 , C14.0, C14.2, C14.8, C76.0, C06.9, C14.8, C32.0, C32.1, C32.3, C32.8, C32.9, C13.0, C13.1, C13.2, C13.8, C13.9, C00.0, C00.1, C00.2, C00.3, C00.4, C00.5, C00.6, C00.8, C00.9, C14.8, C44.0 |
| Hepatic, pancreatic or biliary | C22.0, C22.1, C23, C24, C25 |
| Lung | C34 |
| Lymphoma | C77 |
| Prostate | C61.9 |
| Sarcoma | C00.0, C00.1, C00.3, C00.5, C00.9, C01.9 to C02.3, C02.8 to C03.1, C03.9, C04.0, C04.9, C.05.0, C05.1, C05.9, C06.0, C06.2, C06.9, C07.9, C08.0, C08.9, C09.0, C09.9, C10.3, C10.9, C11.0 to C11.3, C11.8, C11.9, C13.0, C13.1, C13.8, C13.9, C14.0, C14.8, C15.0, C15.3, C15.4, C15.5, C15.9, C16.0 to C16.6, C16.8 to C17.3, C17.8 to C18.9, C19.9, C20.9, C22.0, C22.1, C23.9 to C24.1, C24.9 to C25.2, C25.9, C30.0, C30.1, C31.1 to C31.3, C31.8 to C32.3, C32.9, C33.9 to C34.3, C34.8, C34.9, C37.9 to C38.3, C40.1 to C40.3, C40.8 to C41.4, C41.9, C42.1 to C42.4, C44.0 to C44.9, C47.0 to C47.9, C49.0 to C49.9, C50.0 to C512, C51.8, C51.9, C52.9 to C53.1, C53.8 to C54.3, C54.8, C54.9, C56.9 to C57.4, C57.7 to C57.9, C60.0 to C60.2, C60.9, C61.9 to C62.1, C62.9 to C63.2, C63.7 to C63.9, C649., C65.9, C66.9 to C68.0, C68.8, C69.0, C69.3, C69.6, C69.8, C70.0, C70.1, C70.9 to C72.0, C72.5, C72.9, C73.9 to C74.1, C74.9, C75.5, C77.0 to C77.9 with morphology code 803*, 831*, 871*, 880*-885*, 890*-900*, 912*, 914*, 917*-919*, 922*-924*, 926*, 933*, 944*, 948*, 953*, 958*, 974*-975*, 993* |
| Stomach | C16 |
| Other | C26.0, C26.8, C26.9, C30, C31, C32.2, C33.9, C37.9, C38, C39, C40, C41, C42.0-C42.4, C44.1, C48, C49, C58.9, C63, C76, C80.9 |

*Abbreviations*: CNS, Central nervous system; PNS, Paraneoplastic neurologic syndromes.

**Supplemental Methods**

Regression modeling strategy:

We used a segmented negative binomial regression model with standard parametrization to study the trend in weekly volume of each cancer activity (diagnostic imaging and physician visits) per thousand cancer patients during June 26, 2016-September 26, 2020 (222 weeks):

$$\ln\left( y_{i} \right)=b_{0}+b_{1}*t_{i}+b_{2}*I\left( t_{i}\geq T \right)+b_{3}*I\left( t_{i}\geq T \right)*\left( t_{i}-T \right)+\ln\left( \frac{\mathrm{PatientDay}_{i}}{7} \right)$$

$y_{i}$ = volume of cancer activities in week i, i = 1, 2, …, 222

$t_{i}$ = weekly index (1, 2, …, 222)

$I\left( t_{i}\geq T \right)$ = a dummy variable indicating the pandemic vs. pre-pandemic period, using the index of week March 15-21, 2020 (T) as the start of COVID-19. Hence, this variable = 0 if week i is before the week of March 15-21, 2020 (pre-pandemic period), otherwise equals to 1 (pandemic period).

$\ln\left( \frac{\mathrm{PatientDay}_{i}}{7} \right)$ = offset of the model (denominator of rates = per patient-week)

Hence, the estimated regression parameters b_0_ ~ b_3_ can be interpreted as the following:

$1000*e^{b_{0}}$ = mean cancer activity volume per thousand patients at week 0 (the week before June 26, 2016)

$e^{b_{1}}$= weekly trend over the entire study period (slope), i.e., for each week increase, the volume of cancer activity per thousand patients in that week = $e^{b_{1}}$ * volume per thousand patients in the week prior

$e^{b_{2}}$= rate of change in mean volume per thousand patients at the start of the pandemic period (during the week of March 15, 2020) compared to the previous week.

$e^{b_{3}}$ = change in slope in the pandemic period, i.e., for each week increase during the pandemic, the volume of cancer activity per thousand patients =$e^{b_{1}+b_{3}}*$volume per thousand patients in the week prior

**Supplemental Figure 1** Weekly volume of phone/video visits (red) per thousand cancer patients around the time of cancer diagnosis, June 26, 2016 – September 26, 2020


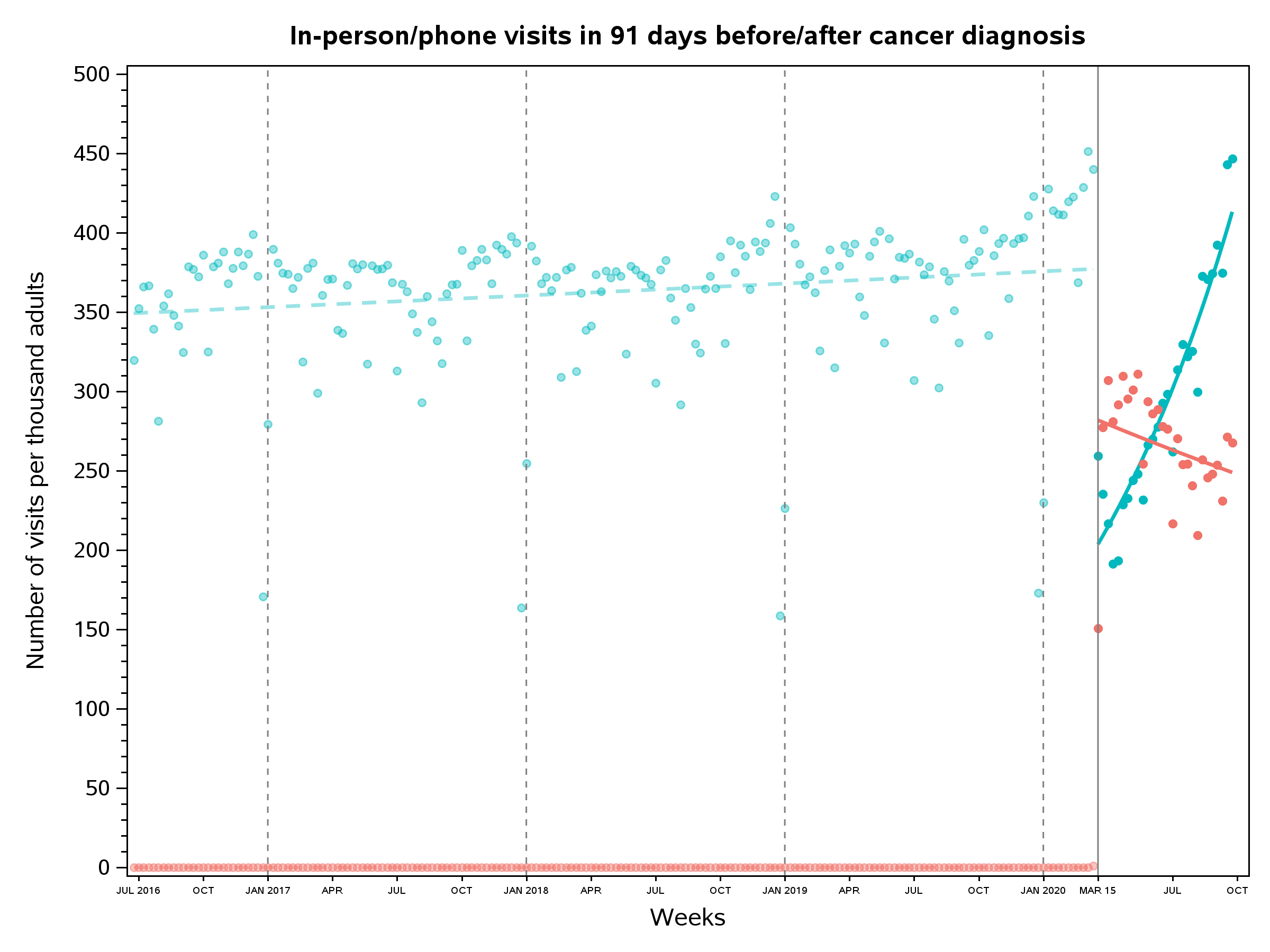


Phone/video visits fee codes (K080, K081, K082, K083) were introduced under a temporary emergency order on March 14, 2020 and are set to expire on September 30, 2022. We did not consider virtual palliative care fee codes (K092, K093, K094, K095) that were launched in March 2021 in the present analysis.

**Supplemental Figure 2** Weekly volume of OTN visits per thousand cancer patients around the date of cancer diagnosis, June 26, 2016 – September 26, 2020


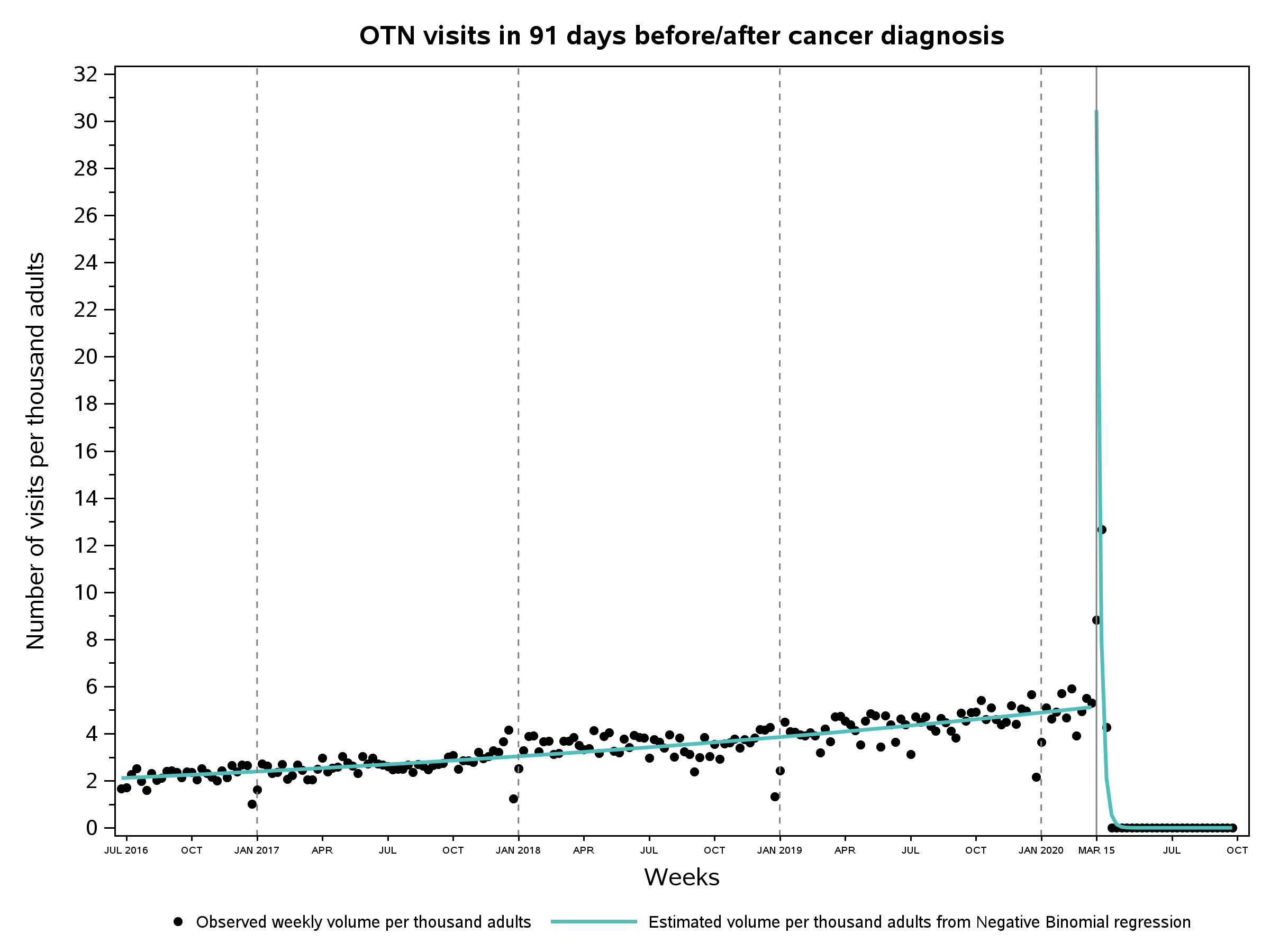


OTN (Ontario Telemedicine Network) visits refer to videoconferencing visits physicians delivered using an OTN platform (i.e., a studio or software that is part of OTN). Until March 31, 2020, physicians could receive a $35 premium for establishing a new patient encounter on OTN and a $15 bonus for each subsequent encounter by billing the B-codes (B100A, B101A, B102A, B200A, B201A, B202A, B099A). These fee codes were discontinued on April 1, 2020, shortly after the introduction of the new temporary codes that allowed physicians to conduct phone/video visits using a non-OTN method (March 14, 2020).

**Supplemental Figure 3** Weekly volume of e-assessment visits per thousand cancer patients around the date of cancer diagnosis, June 26, 2016 – September 26, 2020


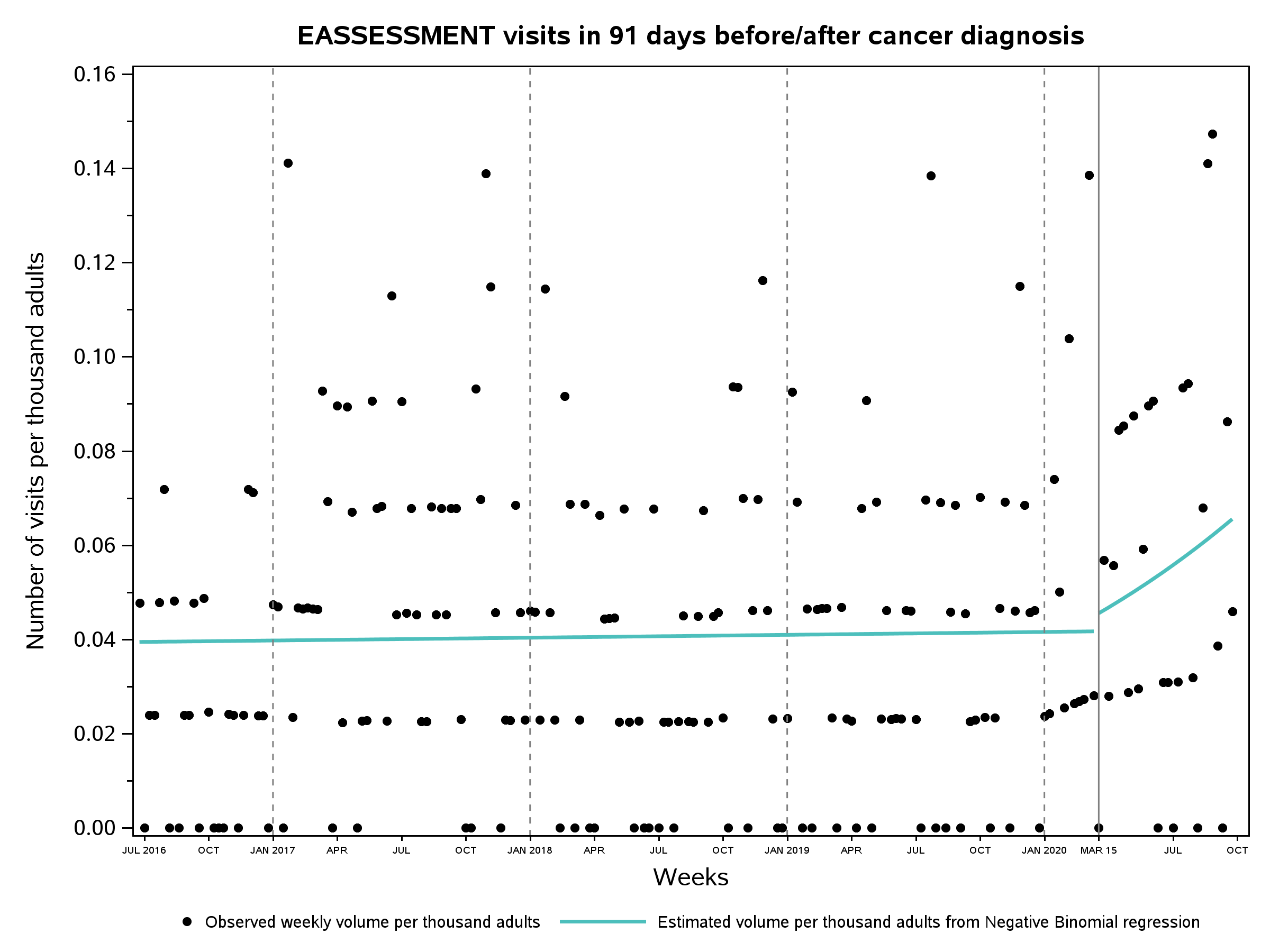


An e-assessment is an electronic reply (such as an email) made by a specialist to respond to a request from a primary care physician or a nurse practitioner on the management of a specific patient.
